# Biogas generation in anaerobic wastewater treatment under tetracycline antibiotic pressure

**DOI:** 10.1038/srep28336

**Published:** 2016-06-24

**Authors:** Meiqing Lu, Xiaojun Niu, Wei Liu, Jun Zhang, Jie Wang, Jia Yang, Wenqi Wang, Zhiquan Yang

**Affiliations:** 1 School of Environment and Energy, South China University of Technology, Guangzhou, 510006, PR China; 2 Guangdong Provincial Key Laboratory of Atmospheric Environment and Pollution Control, Guangzhou, 510640, PR China; 3 State Key Laboratory of Pollution Control and Resource Reuse, Nanjing, 210093, PR China

## Abstract

The effect of tetracycline (TC) antibiotic on biogas generation in anaerobic wastewater treatment was studied. A lab-scale Anaerobic Baffled Reactor (ABR) with three compartments was used. The reactor was operated with synthetic wastewater in the absence of TC and in the presence of 250 μg/L TC for 90 days, respectively. The removal rate of TC, volatile fatty acids (VFAs), biogas compositions (hydrogen (H_2_), methane (CH_4_), carbon dioxide (CO_2_)), and total biogas production in each compartment were monitored in the two operational conditions. Results showed that the removal rate of TC was 14.97–67.97% in the reactor. The presence of TC had a large negative effect on CH_4_ and CO_2_ generation, but appeared to have a positive effect on H_2_ production and VFAs accumulation. This response indicated that the methanogenesis process was sensitive to TC presence, but the acidogenesis process was insensitive. This suggested that the presence of TC had less influence on the degradation of organic matter but had a strong influence on biogas generation. Additionally, the decrease of CH_4_ and CO_2_ generation and the increase of H_2_ and VFAs accumulation suggest a promising strategy to help alleviate global warming and improve resource recovery in an environmentally friendly approach.

Wastewater treatment plants (WWTPs) have become an essential component to ensure necessary water supplies. However, the operation of WWTPs requires a large amount of electricity, up to 0.3–0.8 kWh/m^3 ^1. It has been estimated that wastewater treatment accounts for about 3% of the total US electrical energy load^2^, whereas the corresponding figure in China is 0.3% on average^1^. Consequently, a large amount of fossil fuels are used to produce power to meet this need for electricity, which depletes fossil fuels resources and causes environmental pollution^3^. Therefore, it is necessary to implement energy-saving in wastewater treatment^4^. Anaerobic wastewater treatment is a technology that can meet this goal. With the characteristics of lower power consumption^5^ and great potential to recover renewable energy^6^,^7^, anaerobic wastewater treatment has developed to be a leading suggestion in wastewater treatment^5^.

Though anaerobic wastewater treatment has many advantages compared with other techniques, its adverse environmental impact is inevitable. In the process of anaerobic wastewater treatment, both intermediates and end-products of substrates have a significant impact on the environment. VFAs are intermediates of substrates in anaerobic wastewater treatment. VFAs are good carbon resources for microbes and can be used as raw materials to produce high value-added products in fermentation industry^8^. Additionally, VFAs can enhance electricity generation in microbial fuel cell (MFC)^9^,^10^. The biogas, such as hydrogen(H_2_), methane(CH_4_), and carbon dioxide(CO_2_), are the end-products of substrates in anaerobic wastewater treatment. H_2_ is a clean, recyclable, and efficient energy carrier without adverse environmental impact and potentially plays a key role in sustainable energy usage^11^. Studies have been conducted to improve H_2_ production to generate power to counter part of the power consumption in wastewater treatment^12^,^13^,^14^. CH_4_ is also a renewable energy fuel form^15^, which can be recovered to replace the use of fossil fuels so that fossil fuels resources depletion and environmental pollution can be relieved to some extent^16^. But CH_4_ is also a greenhouse gas. It has been reported that each molecule of CH_4_ causes about 25 times more global warming than a molecule of CO_2_^17^. The emissions of CH_4_ into the atmosphere will have an adverse effect on the environment. Though CH_4_ recovery has been started up in wastewater treatment^6^, its emissions is still large. A recent study reported that the emissions of CH_4_ in wastewater treatment is a major source of CH_4_ in the atmosphere^18^. CO_2_ is another major biogas produced in anaerobic wastewater treatment. It is a well-accepted greenhouse gas and is widely considered to be the main cause of global warming^19^,^20^. A report showed that the CO_2_ emission of a wastewater treatment plant with a daily treatment capacity of 100000 tons almost equals to total annual CO_2_ emissions of 3856 households and 2603 medium-sized cars^21^. In this point of view, the emission of CO_2_ in wastewater treatment greatly contributes to the global warming. Overall, the emission of biogas (H_2_, CH_4_, and CO_2_) in anaerobic wastewater treatment has a significant environmental impact. Therefore, evaluation of the possible biogas generation (H_2_, CH_4_, and CO_2_) in anaerobic wastewater treatment is of great significance.

However, contaminant antibiotics has been frequently detected in aquatic environment^22^. The concentrations of antibiotics range from ng/L to a few μg/L in WWTPs^22^ and a higher level of mg/L can be detected in certain point sources such as hospital and pharmaceutical industry effluents^23^. The tetracycline (TC) group is one of the most frequently detected antibiotics^24^. It is a broad-spectrum active compound, and can inhibit bacterial protein synthesis by binding the 30S ribosomal subunit to prevent the association of the aminoacyl-tRNA to the ribosomal acceptor-A site resulting in a structural change of 16S rRNA^25^. TC is highly sorbable onto clay materials, soil and sediments^26^,^27^. Since TC is easily sorbed onto sewage sludge, it can inhibit the microbes in sludge and alter biogas generation. Therefore, evaluation of the effect of TC on biogas generation in anaerobic wastewater treatment will help to determine the potential bioresource for reuse and the environmental impact of anaerobic wastewater treatment under TC pressure. To date, most studies of TC in wastewater focused on its removal efficiency^27^,^28^,^29^,^30^ and its sorption in sewage sludge^31^,^32^, but there has not been a comprehensive evaluation of biogas generation (H_2_, CH_4_, and CO_2_) in anaerobic wastewater treatment under TC pressure.

As a high-rate anaerobic reactors, the anaerobic baffled reactor (ABR) has become an attractive option for anaerobic wastewater treatment due to its multiple advantages^33^,^34^,^35^,^36^. ABR is compartmentalized with baffles allowing the partial separation of the processes of acidogenesis and methanogenesis longitudinally down the reactor so these processes occur under their favorable conditions^37^, and biogas is efficiently generated.

The purpose of this study was to investigate the biogas generation under 250 μg/L TC in an ABR for a long-term assessment of 90 days. The removal rate of TC, the levels of volatile fatty acids (VFAs), biogas compositions (H_2_, CH_4_, and CO_2_) and total biogas production in each compartment were monitored, and the environmental impact of TC on anaerobic wastewater treatment was evaluated.

## Results

### Fate of tetracycline (TC) during the operation

**Figure 1:**
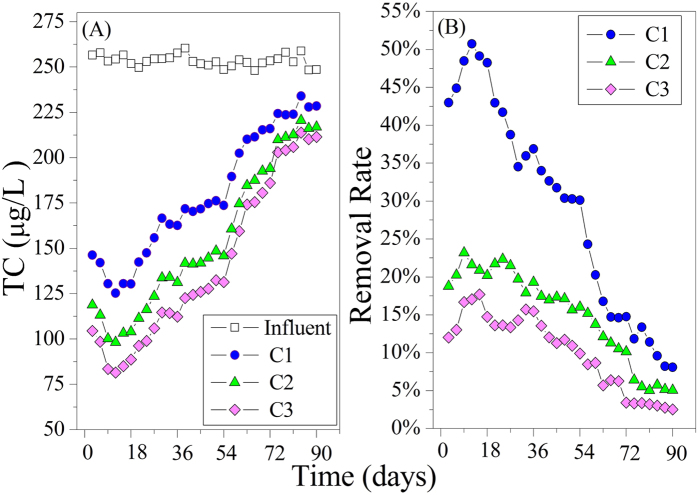
(**A**) Tetracycline concentration in the effluent of each compartment of the reactor. (**B**) The removal rate of TC in each compartment.

TC was measured in the effluent and influent in each compartment in order to ascertain its removal in the reactor. Measurement indicated that TC concentrations in the effluent were always significantly lower than the corresponding influent as seen in Fig. 1. In C1, the removal rate of TC ranged from 8.10–50.74% during operation, and it increased at the beginning of TC exposure. The maximum TC removal rate (50.74%) occurred in the 12th day after TC addition, then it decreased. The rate of this decrease slowed with time. In C2, the removal rate of TC ranged from 5.05–23.18% during operation. This rate also increased in the first few after TC exposure. The maximum TC removal rate (23.18%) occurred the 9^th^ day after TC addition and then it slowly decreased and flattened out by the final 18 days of operation. In C3, the removal rate of TC ranged from 2.52–17.71%, It reached the maximum level (17.71%) on the 15^th^ day, plateaued for a few days, increased slightly days 21–33, and then decreased and flattened out by the final 18 days of operation. Overall, the removal rate of TC was 14.97–67.97% in the reactor.

### Effects of TC on volatile fatty acids (VFAs) in the ABR

**Figure 2:**
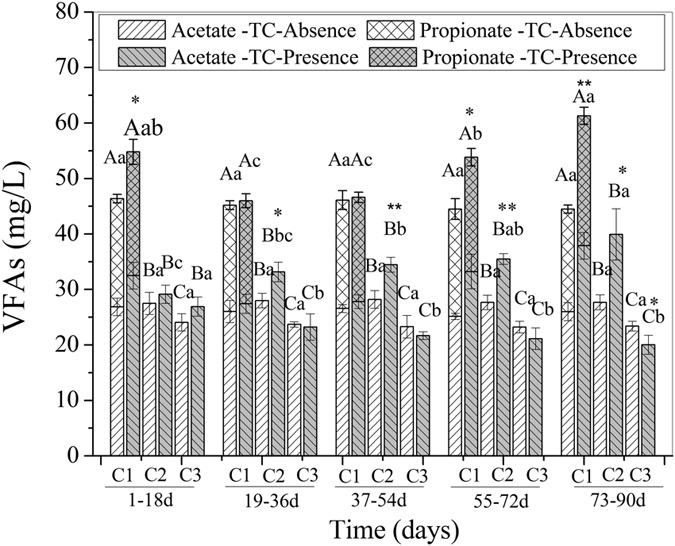
Responses of VFAs in each compartment of the reactor. The data are presented as means ± SD from triplicates for each determination, ANOVA significant at P ≤ 0.05. Separate test the significant value in TC-absence and TC-presence wastewater condition. Different capital letters indicate significantly different values among compartments at each monitored time, and different small letters indicate significantly different values among exposure times in a particular compartment (DMRT, P ≤ 0.05). * and ** indicate the significant value of T-test between TC-absence and TC-presence wastewater operation at P ≤ 0.05 and P ≤ 0.01.

The performance of volatile fatty acids (VFAs) under the two conditions is shown in Fig. 2. The detected VFAs were acetate and propionate, and propionate was detected only in C1. The average concentrations of VFAs in C1, C2, and C3 were 45.33 mg/L, 27.81 mg/L, and 23.53 mg/L, respectively, in the TC-absence wastewater.

The concentration of VFAs were at higher levels with TC exposure than in the absence of TC in C1 and C2: 45.99–54.80 mg/L (C1) and 29.12–39.93 mg/L (C2), corresponding to 1.01–1.35 times and 1.05–1.44 times the average in the TC-absence wastewater. In C3, the response of VFAs was quite different from C1 and C2. In the initial 18 days with TC exposure, the concentration of VFAs was slightly higher than the average in the TC-absence wastewater, and then showed a slight decrease during the operation. The corresponding averages of VFAs were 20.02–26.91 mg/L, which was 85.08–114.36% of the average in the TC-absence wastewater. T-test results showed that TC has a significant effect on VFAs in days 1–18 and days 55–72 in C1, in days 19–36 and days 73–90 in C2, and in days 73–90 in C3, and a very significant effect on VFAs in days 73–90 in C1, and in days 37–54 and days 55–72 in C2.

### Effects of TC on biogas compositions (H_2_, CH_4_, CO_2_) in the ABR

**Figure 3:**
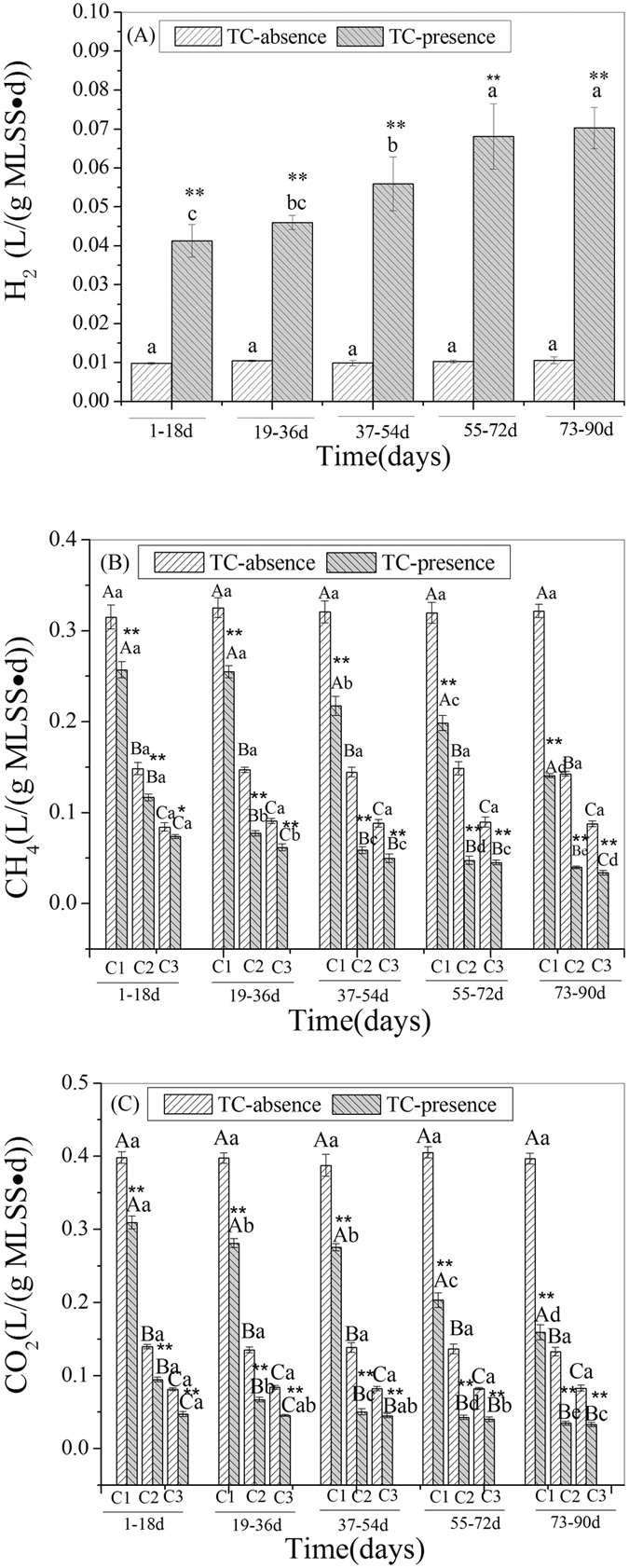
Responses of biogas compositions (H_2_, CH_4_, CO_2_) in each compartment of the reactor: (**A**) H_2_; (**B**) CH_4_; (**C**) CO_2_. The data are presented as means ± SD from triplicates for each determination, ANOVA significant at P ≤ 0.05. Separate test the significant value in TC-absence and TC-presence wastewater condition. Different capital letters indicate significantly different values among compartments at each monitored time, and different small letters indicate significantly different values among exposure times in a particular compartment (DMRT, P ≤ 0.05). * and ** indicate the significant value of T-test between TC-absence and TC-presence wastewater operation at P ≤ 0.05 and P ≤ 0.01.

The performances of gas compositions (H_2_, CH_4_, CO_2_) are shown in Fig. 3. The monitored gases were H_2_, CH_4_, CO_2_, which are the dominant biogas produced in anaerobic wastewater treatment process.

H_2_ was only detected in C1 (Fig. 3A). The generation average of H_2_ in C1 was 0.0102 L/(g MLSS · d) in the TC-absence wastewater. With TC exposure, the generation of H_2_ in C1 showed a sharply increase. The averages H_2_ generation was 0.0413–0.0703 L/(g MLSS · d), which corresponded to 4.05–6.89 times that of the average in the TC-absence wastewater. T-test results showed that TC had a very significant effect on H_2_ generation in C1 during the exposure.

CH_4_ (Fig. 3B) was produced in all compartments in the reactor. In the TC-absence wastewater operation, the average generation of CH_4_ was 0.3204 L/(g MLSS · d), 0.1462 L/(g MLSS · d), and 0.881 L/(g MLSS · d) in C1, C2, and C3, respectively. With TC addition, levels decreased to different extents in the different compartments. The generation of CH_4_ was 0.1407–0.2570 L/(g MLSS d), 0.0398–0.1167 L/(g MLSS d), and 0.0337–0.0737 L/(g MLSS d) in C1, C2, and C3 with TC exposure, which corresponded to 43.90–80.20%, 27.18–79.79%, and 38.24–83.70% of the average in the TC-absence wastewater. T-test results showed that TC had a very significant effect on CH_4_ generation in all compartments during TC exposure except for days 1–18 in C3 (a significant effect).

For CO_2_ (Fig. 3C), the average generation of CO_2_ was 0.3970L/(g MLSS · d), 0.1364L/(g MLSS · d), and 0.0823 L/(g MLSS · d) in C1, C2, and C3 respectively in the operation of TC-absence wastewater. With TC exposure, the generation of CO_2_ decreased to 0.1592–0.3095 L/(g MLSS · d), 0.0349–0.0943 L/(g MLSS · d), and 0.0330–0.0472 L/(g MLSS · d) with TC exposure. That corresponded to 40.10–77.95%, 25.61–69.18%, and 40.07–57.39% of the average in the TC-absence wastewater. T-test results showed that TC had a very significant effect on CO_2_ generation in all compartments during TC exposure.

Obviously, TC enhanced H_2_ production and reduced CH_4_ and CO_2_ production, and the reduction of CH_4_ production was smaller than the effect on CO_2_ production.

### Effects of TC on the total biogas generation in the ABR

**Figure 4:**
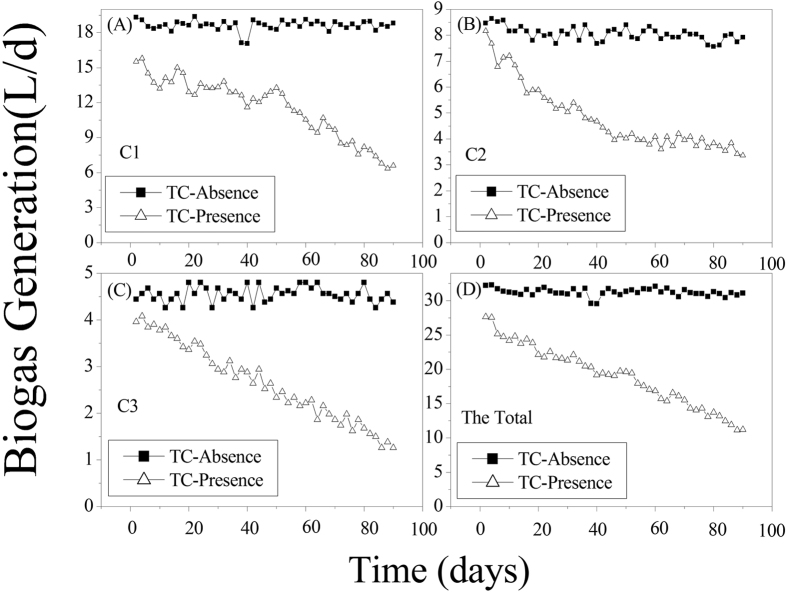
Responses of biogas generation in each compartment of the reactor: (**A**) C1; (**B**) C2; (**C**) C3; (**D**) Total biogas generation in the reactor.

The average total biogas generation in ABR was 31.23 L/d (18.62 L/d, 8.06 L/d, and 4.55 L/d in C1, C2, and C3, respectively) in the TC-absence wastewater while the corresponding average levels were 18.98 L/d (11.54 L/d, 4.81 L/d, and 2.63 L/d in C1, C2, and C3, respectively) in the TC-presence wastewater (Fig. 4). Obviously, TC showed a negative effect on the biogas generation in our study. The total biogas decline rate was 39.23%, a combination of the increased H_2_ production and decreased CH_4_ and CO_2_ production.

## Discussion

Quite different removal rate of TC either in WWTPs^27^,^30^ or Lab-scale experiments have been reported^25^,^31^,^38^,^39^. For instance, a TC removal rate of 67.9–100% was reported by Karthikeyan and Meyer^40^ after secondary wastewater treatment in a WWTP. In the lab-scale experiments, Cetecioglu *et al*.^25^ measured the removal rate of TC as 40–90% in different phases in a anaerobic sequencing batch reactor before metabolic activities practically declined and nearly ceased. Aydin *et al*.^39^ found that TC removal efficiency ranged from 60–95%, and this rate was lower than 3% when the COD removal efficiency and biogas production significantly decline after dosing with an antibiotic mixture (containing TC). Kim *et al*.^31^ reported a TC removal rate of 78.4% in a sequencing batch reactor spiked with 250 μg/L TC, and Matos *et al*.^38^ observed a 28% TC removal rate in a sequencing batch biofilm reactor. In our present study, the overall TC removal rate was 14.97–67.97% in the reactor: 8.10–50.74% of C1, 5.05–23.18% of C2, and 2.52–17.71% of C3. The different results in these studies could be attributed to variations in the anaerobic reactor types, the cultivation and operating conditions or antibiotic combinations and concentrations used in the studies. In our present study, the increase in the TC removal rate at the beginning of TC exposure in all compartments and the temporary fluctuations of TC removal rate in C2 and C3 could be explained by sorption mechanism. When sludge-solution equilibrium was reached, the TC removal rate in the relatively balanced state could be explained by the biodegradation mechanism. As shown in Fig. 1, the TC removal rate in all compartments increased at the beginning of TC exposure because the adsorption capacity of sludge was high and the TC was easily sorbed onto sludge. As sorption continued, the adsorption capacity of sludge decreased, resulting in a decrease in the TC removal rate. The TC removal rate in C2 and C3 declined slower than in C1. This was probably due to the delay of reaching sludge-solution equilibrium in subsequent compartments due to the influenced of the effluent from the prior compartment. The slight increase of TC removal rate in C3 during days 21–33 resulted because the increase of effluent TC concentration from front compartment forced the sludge to absorb a lot of TC to reach a new sludge-solution equilibrium. With longer processing time, TC trended to reach sludge-solution equilibrium in all compartments, and then TC removal rate reached a relatively balanced state. During this time, sorption was in a dynamic balance and had very little to do with TC removal, so biodegradation was responsible for the TC removal in this period. In general, sorption and biodegradation could explain the TC removal rate in our study, and sorption was the main factor, which is consistent with most previous studies^26^.

VFAs were intermediate of substrates in the acidification processes of anaerobic biodegradation, and resulting in the eventual production of H_2_, CH_4_ and CO_2_. The determination of VFAs is an essential prerequisite to determine the impact of TC on metabolic activities under anaerobic conditions. In our present study, the detected VFAs were acetate and propionate, and propionate was only detected in C1. This indicated that the fermentation pattern in the reactor was typical of propionic-type fermentation, and the conversations of propionate to acetate was more efficient in C2 and C3. As seen in Fig. 2, the concentrations of VFAs in C1, C2, and C3 on average were 45.33 mg/L, 27.81 mg/L, and 23.53 mg/L, respectively, in the TC-absence wastewater. Obviously, VFAs concentration decreased longitudinally down the reactor due to the compartmentalization of the active processes of acidification and methanogenesis, VFAs were produced during the acidification, so the front compartment would likely generate more VFAs. Additionally, the available substrates for acidification decreased and the efficient conversations of VFAs to CH_4_ and CO_2_ increased longitudinally down the reactor. That is consistent with the previous studies^33^,^36^. With TC exposure, the concentration of VFAs in C1 and C2 corresponded to 1.01–1.35 times and 1.05–1.44 times higher than that of the average in the TC-absence wastewater. Similar findings were reported by Aydin *et al*.^41^ who found that feeding an antibiotic mixture (containing TC) feeding in an anaerobic sequencing batch reactor showed a greater accumulation of VFAs during anaerobic digestion compared with a no-antimicrobial control. Stone *et al*.^42^ also reported that the concentration of VFAs increased in the presence of oxytetracycline (a kind of TC antibiotic) during operation of an anaerobic digester. The accumulation of VFAs resulted from change in the dynamic balance of generation from glucose acidification by acetogens and H_2_/CO_2_ conversion by homoacetogenic bacteria, and the utilization of acetate by aceticlastic methanogens to produce CH_4_ under anaerobic conditions^16^. There are three reasons for the increased VFAs accumulation observed in our study. First, acetate conversion would be inhibited because the presence of the antibiotic could have a dramatic effect on aceticlastic methanogens^43^,^44^, this is consistent with the decrease of CH_4_ production shown in Fig. 3B. Second, the conversion of H_2_/CO_2_ by homoacetogenic bacteria to acetate might be promoted because more H_2_ (Fig. 3A) was available with TC exposure, but this process required certain conditions and thus only made a small contribution^15^. Third, COD degradation efficiency was promoted in the TC-presence wastewater (see Supplementary Information Figure S1), which indicated increased VFAs generation. That further suggested that acetogens could survive and proliferatee under these conditions because sensitive species were inhibited, as demonstrated by faster growth kinetics and a better adaption rate of antibiotics^45^. Additionally, the temporary fluctuations of VFAs concentration matched the fluctuations of COD degradation (see Supplementary Information Figure S1), suggesting which illustrated that glucose acidification played a role in the observed change in VFAs concentration. In C3, the response of VFAs was quite different from that of C1 and C2 and the concentration of VFAs in the TC-presence wastewater was 85.08–114.36% of the average in the TC-absence wastewater. Except for the initial 18 days of TC exposure, the concentration of VFAs was lower than the average in the TC-absence wastewater during operation. The reasons were illustrated as follows. Glucose acidification was promoted in the initial 18 days (see Supplementary Information Figure S1), which could explain the temporarily higher level of VFAs concentration in the TC-presence wastewater than in the TC-absence wastewater during that time. With time increased, glucose acidification was inhibited (see Supplementary Information Figure S1) and the VFAs generation was decreased, which was mainly due to the inhibition of acetogens. C3 was a weak zone of acidification and acetogens were vulnerable to TC inhibition under its continuous stress. Though VFAs accumulation can be enhanced by the inhibition of the conversion of VFAs to CH_4_ by aceticlastic methanogens, the inhibition generation from glucose acidification by acetogens caused a decrease in VFAs, resulting in a lower concentration of VFAs in TC-presence wastewater treatment.

H_2_ was measured in C1, but it was not detected in C2 and C3. A similar was reported by Ban *et al*.^33^ who detected H_2_ only in the front few compartments of ABR. In the ABR, the active zone of acidogenesis and methanogenesis are longitudinally down the reactor. C1 was an active acidification zone and H_2_ was effectively produced. C2 and C3 show increasing active methanogenesis, in which H_2_ can effectively transfer to CH_4_ by hydrogenotrophic methanogens. The measured H_2_ was significantly promoted in C1 with TC exposure (Fig. 3A), reaching levels 4.05–6.89 times of the average in TC-absence wastewater. H_2_ is generated from glucose acidification by acetogens, and homoacetogenic bacteria or hydrogenotrophic methanogenes can utilize H_2_ to convert acetate or CH_4_. As discussed in the VFAs section, glucose acidification was more efficient in the presence of TC in C1 (see Supplementary Information Figure S1), which could enhance H_2_ generation. Additionally, hydrogenotrophic methanogens were inhibited under TC pressure^44^, which reduced H_2_ consumption and thus resulting in an increase in H_2_ accumulation.

CH_4_ is the terminal end-product of glucose anaerobic digestion. Aceticlastic methanogens and hydrogentrophic methanogens are primarily responsible for this process. Aceticlastic methanogens transform acetate to CH_4_ (about 70% of the total CH_4_) and hydrogentrophic methanogens can utilize H_2_/CO_2_ to produce CH_4_ (about 30% of the total CH_4_)^46^,^47^. The production of CH_4_ in the TC-presence wastewater in C1, C2, and C3 corresponded to 43.90–80.20%, 27.18–79.79%, and 38.24–83.70%, respectively, that of the average in the TC-absence wastewater. TC reduced the production of CH_4_ in all compartments. Similarly, a 25% reduction in CH_4_ generation by the presence of TC was reported in swine manure sequencing batch reactors^48^ and a 50% reduction in CH_4_ generation was reported in an anaerobic digestion in the presence of chlortetracycline (a kind of tetracycline antibiotic)^49^. The inhibition of both aceticlastic methanogens and hydrogentrophic methanogens under TC pressure likely explains the decrease of CH_4_ production and the increase in VFAs (Fig. 2) and H_2_ (Fig. 3A) accumulation in our study resulted from this inhibition. This is consistent previous studies which suggested that methanogens are more sensitive to changes than other microorganisms under antibiotic pressure in anaerobic digestion^50^,^51^.

Glucose acidification by acetogens and acetate conversion by aceticlastic methanogens are the two pathways for CO_2_ generation in anaerobic condition. The utilization of CO_2_ (by combining with H_2_) to convert CH_4_ (hydrogentrophic methanogens) or acetate (homoacetogenic bacteria) also affect the CO_2_ accumulation. In our study, CO_2_ production in C1, C2, and C3 in the TC-presence wastewater corresponded to 40.10–77.95%, 25.61–69.18%, and 40.07–57.39% of the average in the TC-absence wastewater. Obviously, TC reduced CO_2_ production, similar to the findings of Stone *et al*.^32^ who reported that chlortetracycline (a kind of tetracycline antibiotic) resulted in a 28.4% reduction of CO_2_ production during batch anaerobic swine manure digestion. Aceticlastic methanogens and hydrogentrophic methanogens were both inhibited under TC pressure, but inhibition of these organisms showed different effects on CO_2_ generation^50^,^51^. The inhibition of aceticlastic methanogens resulted in the decreased CO_2_ generation but the inhibition of hydrogenotrophic methanogens exerted a positive effect on CO_2_ accumulation (reduced CO_2_ consumption). Overall, hydrogenotrophic methanogens were less sensitive than aceticlastic methanogens^52^,^53^ and the accumulation of CO_2_ from the inhibited H_2_/CO_2_ conversion (hydrogenotrophic methanogens) made a smaller to CO_2_ accumulation^13^. The process of H_2_/CO_2_ conversion to acetate (homoacetogenic bacteria) only occurred under certain conditions similarly played only a small part in CO_2_ accumulation^15^. As the changes of hydrogenotrophic methanogens and homoacetogenic bacteria groups only modestly affect CO_2_ consumption. Therefore, the inhibition of aceticlastic methanogens was the best explanation for the decrease in CO_2_ production.

Overall, the TC removal rate was 14.97–67.97% in the reactor. TC had a large negative effect on CH_4_ and CO_2_ generation, which caused a large decrease of total biogas production. However, the presence of TC apparently had a positive effect on H_2_ and VFAs accumulation. All this responses indicated that the methanogenesis process was sensitive to TC presence, but the acidogenesis process was insensitive. This suggests that the presence of TC had less influence on the degradation of organic matter but had a strong influence on CH_4_ and CO_2_ generation. The reduced CH_4_ and CO_2_ production is beneficial for emission reduction in anaerobic wastewater treatment, which can alleviate the greenhouse effect and global warming. Additionally, additional electricity can be produced from the additional H_2_ and VFAs accumulation, which can help reduce the use of fossil fuels. Also, VFAs can be used as raw materials for production of higher added value fermentation products. Overall, the promoted H_2_ and VFAs accumulation can provide higher added values for resource recovery^54^,^55^,^56^,^57^, which is good for energy-saving. Regardless of the inhibition of mechanism, the presence of TC in wastewater provides a new point of view for energy-saving and emission reduction in anaerobic wastewater treatment. Therefore, methods can be used in anaerobic wastewater treatment to avoid the negative effects of TC and benefit from TC’s positive effects.

## Methods

### Bioreactor System

A lab-scale Anaerobic Baffled Reactor (ABR) (455 × 150 × 400 mm, LWH) with a working volume of 21.0 L was employed for the investigation. It was separated into three compartments (named as C1, C2, C3 in sequence) with a volume ratio of 1.5:1:1, and each compartment was further divided into down-flow and up-flow sections, with a volume ratio of 1:3 by vertical baffles. A bottom edge slanted at 45° produced effective mixing and intimate contact between the wastewater and anaerobic sludge. Each compartment was set with two sample outlets (the upper one for water sample collection and the bottom one for sludge sample collection) on the side of up-flow section and one gas collection outlet on the center of the upper section. The reactor operated at 35(±1) °C in a dark environment in a calorstat and the feed was pumped into the reactor by a peristaltic pump. The evolved biogas was collected from the gas collection outlet of each compartment and was daily measured by wet gas meters (Model LML-1, Changchun Filter Co, Ltd).

### Seed sludge

The reactor was seeded with anaerobically sewage sludge taken from the anaerobic reactor of Lijiao municipal sewage treatment plant (Guangzhou, China), which applied anaerobic-anoxic-oxic technology to the treatment. It was sieved (0.3 mm) to remove debris and large particles at first, and then introduced into compartments of one third volume of each compartment. The characteristics of inoculated sludge are described as follow: MLSS (g·L^−1^), 24.67, MLVSS/MLSS (%), 61.33, pH, 6.58, moisture content (%), 97.3%.

After seeding, the reactor was sealed, and the head space above each compartment was flushed with nitrogen gas to remove residual air from the system^36^.

### Reactor started up and operating conditions

The system was started up at a hydraulic retention time of 24 h with an initial influent COD of 500 mg·L^−1^ and then was increased to 2,000 mg·L^−1^ stepwise. And the system was fed by synthetic wastewater composed of glucose, ammonium chloride and a number of nutrients and trace elements in order to provide a balanced feed to the reactors. The composition of synthetic wastewater was as follows (in g·L^−1^): Glucose, 2; NH_4_Cl, 0.032; KH_2_PO_4_, 0.1316, FeSO_4_·7H_2_O, 0.014, MgSO_4_, 0.248. Additionally, trace elements solution were used 1 milliliter per litre of synthetic wastewater, the composition of trace elements solution were as follows (in mg·L^−1^): CaCl_2_·2H_2_O, 330, CoCl_2_·6H_2_O, 240, MnCl_2_·4H_2_O, 990, NH_4_MoO_4_·4H_2_O, 9, NiCl_2_·6H_2_O, 190, EDTA, 5000, H_3_BO_4_, 14, ZnSO_4_·7H_2_O, 430, CuSO_4_·5H_2_O, 250^35^. NaHCO_3_ was added to make an influent pH value of 7.5 ± 0.1. During an initial start-up period of 56 days, the ABR got stabilized. After stabilization, the reactor was operated in the synthetic wastewater in the presence and absence of TC for 90 days, respectively.

### Analytical methods

The reactor was monitored daily for total biogas generation, COD (chemical oxygen demand). Gas compositions (H_2_, CH_4_, CO_2_), volatile fatty acids (VFAs), MLSS (mixed liquor suspended solids) were measured every 6 days, and the soluble TC concentration were measured every 3 days.

Total biogas generation was measured by the wet gas meter (Model LML-1, Changchun Filter Co, Ltd). COD, and MLSS were determined according to the Standard Methods^58^.

Biogas compositions (H_2_, CH_4_, CO_2_) and VFAs were analyzed by a gas chromatograph (Agilent Technologies 7820 A, USA) equipped with a thermal conductivity detector (TCD) and a flame ionization detector (FID). Biogas compositions (H_2_, CH_4_, CO_2_) were determined by the thermal conductivity detector (TCD) equipped with stainless steel column (2 m × 3.2 mm) packed with TDX-01 (80/100 mesh)^59^, the operational temperatures of injector, oven and detector were kept at 100, 100, and 200 °C, respectively. N_2_ was used as a carrier gas at a flow rate of 35 mL/min. The sample injection volume was 1 mL. Volatile fatty acids (VFAs) were analyzed in the soluble phase by using the flame ionization detector (FID) equipped with a HP-FFAP capillary column (30 m × 0.32 mm internal diameter)^60^. The operational temperatures of injector and detector were kept at 220 °C and 250 °C, respectively. The oven temperature programme started at 90 °C and after 3 min was increased by 20 °C/min to 120 °C and then held for 6 min. N_2_ was used as a carrier gas at a flow rate of 40 mL/min. The sample injection volume was 1 μL.

SPE (Oasis HLB cartridge, 500 mg, 6 mL, Waters, Milford, MA) and HPLC-MS/MS (Agilent 1200 Series, Agilent QQQ 6410, USA) were used to analyze soluble concentrations of TC. An Agilent XDB-C18 analytical column (2.1 × 150 mm, 3.5 μm) was used, and the column temperature was 23 °C. Solvents B and C were acetonitrile and 0.2% formic acid in nanopure water, and the flow rate was 0.4 mL/min. The percentage of solvent B and C were 15% and 85%, respectively. The sample injection volume was 3 μL. TC were ionized in the positive ionization mode and analyzed with the following parameters: capillary voltage 4000 V; nebulizer pressure 35 psi; drying gas, N_2_, 8 L/min; gas temperature 350 °C.

### Statistical analysis

Average and standard deviation values were calculated according to standard procedures. Two-way analysis of variance (ANOVA), i.e. Duncan’s multiple range test (DMRT) and T-test, were used to compare mean values and to assess the significance of the differences between mean values. All statistical analyses were carried out using the SPSS statistical software, version 19.0.

## Additional Information

**How to cite this article**: Lu, M. *et al*. Biogas generation in anaerobic wastewater treatment under tetracycline antibiotic pressure. *Sci. Rep*. **6**, 28336; doi: 10.1038/srep28336 (2016).

## Supplementary Material

Supplementary Information

## References

[b1] Hao X., Liu R. & Huang X. Evaluation of the potential for operating carbon neutral WWTPs in China. Water Res. 87, 424–431 (2015). 26072280 10.1016/j.watres.2015.05.050

[b2] USEPA. Wastewater Management Fact Sheet: Energy Conservation. Office of Water. (2006). Available at: http://www.docin.com/p-513193224.html. (Accessed: 4th November 2015).

[b3] Shafiee S. & Topal E. When will fossil fuel reserves be diminished? Energ Policy 37, 181–189 (2009).

[b4] Wang K. & Liu Y. N. Prospect of China’s energy conservation and emission reduction during the remaining years of the 12th Five-Year Plan period. International Journal of Global Energy Issues 39, 18–34 (2016).

[b5] Tauseef S. M., Abbasi T. & Abbasi S. A. Energy recovery from wastewaters with high-rate anaerobic digesters. Renew. Sust. Energ. Rev. 19, 704–741 (2013).

[b6] McCarty P. L., Bae J. & Kim J. Domestic wastewater treatment as a net energy producer–can this be achieved? Environ Sci Technol. 45, 7100–7106 (2011). 21749111 10.1021/es2014264

[b7] Zang Y. . Towards more accurate life cycle assessment of biological wastewater treatment plants: a review. Journal of Cleaner Production 107, 676–692 (2015).

[b8] Kim H., Kim J. & Shin S. G. Continuous fermentation of food waste leachate for the production of volatile fatty acids and potential as a denitrification carbon source. Bioresour. Technol. 207, 440–445 (2016). 26922002 10.1016/j.biortech.2016.02.063

[b9] Liu H., Cheng S. & Logan B. E. Production of electricity from acetate or butyrate using a single-chamber microbial fuel cell. Environ Sci Technol. 39, 658–662 (2005). 15707069 10.1021/es048927c

[b10] Min B. & Logan B. E. Continuous electricity generation from domestic wastewater and organic substrates in a flat plate microbial fuel cell. Environ Sci Technol. 38, 5809–5814 (2004). 15575304 10.1021/es0491026

[b11] De Gioannis G., Muntoni A., Polettini A. & Pomi R. A review of dark fermentative hydrogen production from biodegradable municipal waste fractions. Waste Manag 33, 1345–1361 (2013). 23558084 10.1016/j.wasman.2013.02.019

[b12] Ismail I., Hassan M. A., Rahman N. A. A. & Soon C. S. Thermophilic biohydroge production from palm oil mill effluent (POME) using suspended mixed culture. Biomass Bioenergy 34, 42–47 (2010).

[b13] Kim D. H., Lee M. K., Jung K. W. & Kim M. S. Alkali-treated sewage sludge as a seeding source for hydrogen fermentation of food waste leachate. *Int. J*. Hydrogen Energy 38, 15751–15756 (2013).

[b14] Sydney E. B. . Economic process to produce biohydrogen an volatile fatty acids by a mixed culture using vinasse from sugarcane ethanol industry as nutrient source. Bioresour. Technol. 159, 5140–5144 (2014). 10.1016/j.biortech.2014.02.042 24675397

[b15] Demirel B. & Scherer P. The roles of acetotrophic and hydrogenotrophic methanogens during anaerobic conversion of biomass to methane: a review. Rev. Environ. Sci. Bio/Technol 7, 173–190 (2008).

[b16] Chynoweth D. P., Owens J. M. & Legrand R. Renewable methane from anaerobic digestion of biomass. Renew Energy 22, 1–8 (2001).

[b17] IPCC (Intergovernmental Panel on Climate Change). Climate change 2007: working group III: mitigation of climate change. IPCC, Paris (2007).

[b18] Abbasi T., Tauseef S. M. & Abbasi S. A. Anaerobic digestion for global warming control and energy generation—An overview. Renew. Sust. Energ. Rev. 16, 3228–3242 (2012).

[b19] Galagan J. E. . The genome of M. acetivorans reveals extensive metabolic and physiological diversity. Genome Res. 12, 532–542 (2002). 11932238 10.1101/gr.223902 PMC187521

[b20] Royer D. L., Berner R. A. & Park J. Climate sensitivity constrained by CO_2_ concentrations over the past 420 million years. Nature 446, 530–532 (2007). 17392784 10.1038/nature05699

[b21] Huang H. The Significance and prospects of water treatment technology in sewage processing. China New Technologies and Products 08, 57 (2011) (in Chinese).

[b22] Kümmerer K. Antibiotics in the aquatic environment – a Review – Part I. Chemosphere 75, 417–434 (2009). 19185900 10.1016/j.chemosphere.2008.11.086

[b23] Marathe N. P. . A treatment plant receiving waste water from multiple bulk drug manufacturers is a reservoir for highly multi-drug resistant integron-bearing bacteria. PLoS One 8, e77310 (2013). 24204801 10.1371/journal.pone.0077310 PMC3812170

[b24] Watkinson A. J., Murby E. J. & Costanzo S. D. Removal of antibiotics in conventional and advanced wastewater treatment: implications for environmental discharge and wastewater recycling. Water Res. 41, 4164–4176 (2007). 17524445 10.1016/j.watres.2007.04.005

[b25] Cetecioglu Z. . Chronic impact of tetracycline on the biodegradation of an organic substrate mixture under anaerobic conditions. Water Res. 47, 2959–2969 (2013). 23561494 10.1016/j.watres.2013.02.053

[b26] Li B. & Zhang T. Biodegradation and adsorption of antibiotics in the activated sludge process. Environ Sci Technol. 44, 3468–3473 (2010). 20384353 10.1021/es903490h

[b27] Michael I. . Urban wastewater treatment plants as hotspots for the release of antibiotics in the environment: a review. Water Res. 47, 957–995 (2013). 23266388 10.1016/j.watres.2012.11.027

[b28] Verlicchi P., Al Aukidy M. & Zambello E. Occurrence of pharmaceutical compounds in urban wastewater: removal, mass load and environmental risk after a secondary treatment—a review. Sci Total Environ. 429, 123–155 (2012). 22583809 10.1016/j.scitotenv.2012.04.028

[b29] Li B. & Zhang T. Mass flows and removal of antibiotics in two municipal wastewater treatment plants. Chemosphere 83, 1284–1289 (2011). 21439606 10.1016/j.chemosphere.2011.03.002

[b30] Le-Minh N., Khan S. J., Drewes J. E. & Stuetz R. M. Fate of antibiotics during municipal water recycling treatment processes. Water Res. 44, 4295–4323 (2010). 20619433 10.1016/j.watres.2010.06.020

[b31] Kim S. . Removal of antibiotics in wastewater: effect of hydraulic and solid retention times on the fate of tetracycline in the activated sludge process. Environ Sci Technol. 39, 5816–5823 (2005). 16124320 10.1021/es050006u

[b32] Prado N., Ochoa J. & Amrane A. Biodegradation and biosorption of tetracycline and tylosin antibiotics in activated sludge system. Process Biochem. 44, 1302–1306 (2009).

[b33] Ban Q. . Linking performance with microbial community characteristics in an anaerobic baffled reactor. Appl Biochem Biotech. 169, 1822–1836 (2013). 10.1007/s12010-013-0105-6 23344944

[b34] Bodkhe S. Y. A modified anaerobic baffled reactor for municipal wastewater treatment. J Environ Manage 90, 2488–2493 (2009). 19233545 10.1016/j.jenvman.2009.01.007

[b35] Gopala Krishna G. V. T., Kumar P. & Kumar P. Treatment of low-strength soluble wastewater using an anaerobic baffled reactor (ABR). J Environ Manage 90, 166–176 (2009). 18096298 10.1016/j.jenvman.2007.08.017

[b36] Uyanik S., Sallis P. J. & Anderson G. K. The effect of polymer addition on granulation in an anaerobic baffled reactor (ABR). Part I: process performance. Water Res. 36, 933–943 (2002). 11848364 10.1016/s0043-1354(01)00315-3

[b37] Barber W. P. & Stuckey D. C. The use of the anaerobic baffled reactor (ABR) for wastewater treatment: a review. Water Res. 33, 1559–1578 (1999).

[b38] Matos M. . Influence of tetracycline on the microbial community composition and activity of nitrifying biofilms. Chemosphere 117, 295–302 (2014). 25127228 10.1016/j.chemosphere.2014.06.094

[b39] Aydin S. . Combined effect of erythromycin, tetracycline and sulfamethoxazole on performance of anaerobic sequencing batch reactors. Bioresour. Technol. 186, 207–214 (2015). 25817031 10.1016/j.biortech.2015.03.043

[b40] Karthikeyan K. G. & Meyer M. T. Occurrence of antibiotics in wastewater treatment facilities in Wisconsin, USA. Sci Total Environ. 361, 196–207 (2006). 16091289 10.1016/j.scitotenv.2005.06.030

[b41] Aydin S. . Inhibitory effects of antibiotic combinations on syntrophic bacteria, homoacetogens and methanogens. Chemosphere 120, 515–520 (2015). 25290357 10.1016/j.chemosphere.2014.09.045

[b42] Stone J. J. . Effect of antimicrobial compounds tylosin and chlortetracycline during batch anaerobic swine manure digestion. Water Res. 43, 4740–4750 (2009). 19695662 10.1016/j.watres.2009.08.005

[b43] Aydin S. . Use of PCR-DGGE based molecular methods to assessment of microbial diversity during anaerobic treatment of antibiotic combinations. Bioresour. Technol. 192, 735–740 (2015). 26101963 10.1016/j.biortech.2015.05.086

[b44] Aydin S., Ince B. & Ince O. Application of Real-Time PCR to determination of combined effect of antibiotics on Bacteria, Methanogenic Archaea, Archaea in anaerobic sequencing batch reactors. Water Res. 76, 88–98 (2015). 25792437 10.1016/j.watres.2015.02.043

[b45] Ma J. . A simple methodology for rate-limiting step determination for anaerobic digestion of complex substrates and effect of microbial community ratio. Bioresour. Technol. 134, 391–395 (2013). 23489573 10.1016/j.biortech.2013.02.014

[b46] Ince B. . Inhibition effect of isopropanol on acetyl-CoA synthetase expression level of acetoclastic methanogen, Methanosaeta concilii. J. Biotechnol. 156, 95–99 (2011). 21884734 10.1016/j.jbiotec.2011.08.021

[b47] Kim J., Lim J. & Lee C. Quantitative real-time PCR approaches for microbial community studies in wastewater treatment systems: applications and considerations. Biotechnol. Adv. 31, 1358–1373 (2013). 23747590 10.1016/j.biotechadv.2013.05.010

[b48] Masse D. I. & Droste R. L. Comprehensive model of anaerobic digestion of swine manure slurry in a sequencing batch reactor. Water Res. 34, 3087–3106 (2000).

[b49] Sanz J. L., Rodriguez N. & Amils R. The action of antibiotics on the anaerobic digestion process. Appl Microbiol Biot. 46, 587–592 (1996). 10.1007/s002530050865 9008891

[b50] Yu Y., Kim J. & Hwang S. Use of real-time PCR for group specific quantification of aceticlastic methanogens in anaerobic processes: population dynamics and community structures. Biotechnol. Bioeng. 93, 424–433 (2006). 16196054 10.1002/bit.20724

[b51] Ozbayram E. G. . Acute effects of various antibiotic combinations on acetoclastic methanogenic activity. Environ. Sci. Pollut. Res. 22, 6230–6235 (2014). 10.1007/s11356-014-3841-4 25408075

[b52] Leaphart A. B. & Lovell C. R. Recovery and analysis of formyltetrahydrofolate synthetase gene sequences from natural populations of acetogenic bacteria. Appl. Environ. Microbiol. 67, 1392–1395 (2001). 11229939 10.1128/AEM.67.3.1392-1395.2001 PMC92742

[b53] Xu K., Liu H., Du G., Chen J. & Real-time P. C. R. assays targeting formyltetrahydrofolate synthetase gene to enumerate acetogens in natural and engineered environments. Anaerobe 15, 204–213 (2009). 19328859 10.1016/j.anaerobe.2009.03.005

[b54] Anderson R. C. . Effect of oral nitroethane and 2-nitropropanol administration on methane-producing activity and volatile fatty acid production in the ovine rumen. Bioresour. Technol. 97, 2421–2426 (2006). 16303299 10.1016/j.biortech.2005.10.013

[b55] Božic A. K. . Effects of the methane-Inhibitors nitrate, nitroethane, lauric Acid, Lauricidin and the Hawaiian marine algae Chaetoceros on ruminal fermentation in vitro. Bioresour. Technol. 100, 4017–4025 (2009). 19362827 10.1016/j.biortech.2008.12.061

[b56] Xu K., Liu H. & Chen J. Effect of classic methanogenic inhibitors on the quantity and diversity of archaeal community and the reductive homoacetogenic activity during the process of anaerobic sludge digestion. Bioresour. Technol. 101, 2600–2607 (2010). 19939675 10.1016/j.biortech.2009.10.059

[b57] Michael K. R. Production of acetic acid from waste biomass. [Ph.D. Thesis]. Texas A&M University (1998).

[b58] APHA, USA. Standard Methods for the Examination of Water and Wastewater (twenty first ed.). American Public Health Association/American Water Works Association/Water Environment Federation, Washington, DC (2005).

[b59] Liu Q. . Phosphate enhancing fermentative hydrogen production from substrate with municipal solid waste composting leachate as a nutrient. Bioresour. Technol. 190, 431–437 (2015). 25739998 10.1016/j.biortech.2015.01.139

[b60] Zhang F. . Fatty acids production from hydrogen and carbon dioxide by mixed culture in the membrane biofilm reactor. Water Res. 47, 6122–6129 (2013). 23941982 10.1016/j.watres.2013.07.033

