# Supplementary material for: Biogas generation in anaerobic wastewater treatment under tetracycline antibiotic pressure
Source: Sci Rep. 2016 Jun 24;6:28336. doi: 10.1038/srep28336 (PMC4920035; doi:10.1038/srep28336)
Supplement: Supplementary Information [file srep28336-s1.pdf]

Supplementary Information for

Biogas generation in anaerobic wastewater treatment under tetracycline antibiotic pressure

Meiqing Lu[a,b], Xiaojun Niu[a,b,c,*], Wei Liu[a,b], Jun Zhang[a,b], Jie Wang[a,b], Jia Yang[a,b], Wenqi Wang[a,b], Zhiquan Yang[a,b]

[a] School of Environment and Energy, South China University of Technology, Guangzhou, 510006, PR China

[b] Guangdong Provincial Key Laboratory of Atmospheric Environment and Pollution Control，Guangzhou, 510640, PR China

[c] State Key Laboratory of Pollution Control and Resource Reuse , ,Nanjing, 210093,　PR China

Supporting figures

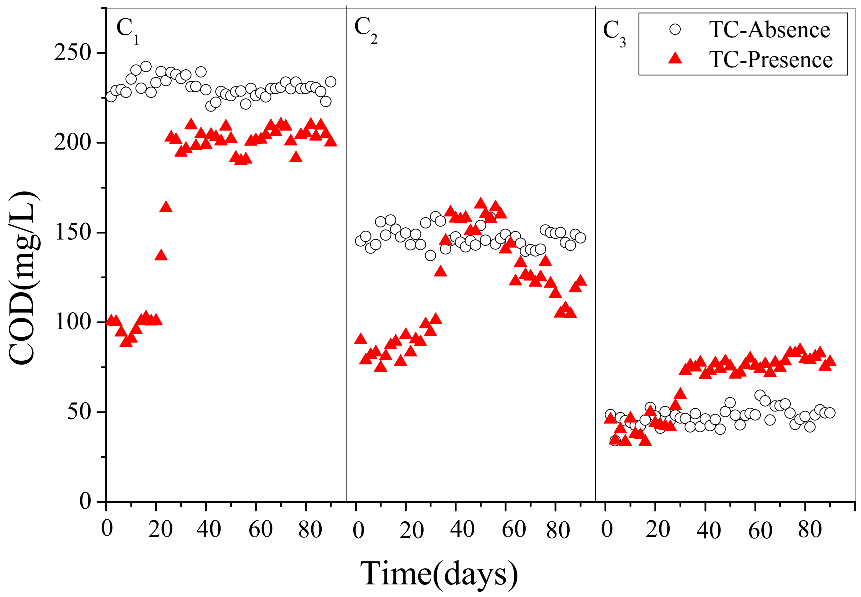

Figure S1 | Responses of effluent COD in each compartment of the reactor.
